# Letter to the Editor: Methodological Concerns Regarding “BCR in Heart Failure: When the Abstract Outruns the Evidence”

**DOI:** 10.1002/clc.70431

**Published:** 2026-07-27

**Authors:** Jobab Nadeem, Hafisba Afzal, Own Muhammad, Aima Rizwan

**Affiliations:** ^1^ International European University Kyiv City Ukraine; ^2^ Azra Naheed Medical College Lahore Pakistan; ^3^ The Superior University Lahore Pakistan; ^4^ Abwa Medical College Faisalabad Pakistan; ^5^ University of Health Sciences Lahore Pakistan

To the Editor,

Re: Cabanillas‐Lazo et al., systematic review of BCR in heart failure [1].

Cabanillas‐Lazo et al. deserve credit for tackling the prognostic value of the blood urea nitrogen‐to‐creatinine ratio (BCR) in heart failure. Twenty cohort studies, over 21 000 patients, a registered protocol, dual quality appraisal. This is a serious effort on a marker that's cheap, accessible, and biologically plausible. Still, we think the abstract drifts from what the full text actually supports, in four ways.

The analytical plan changed, and nobody mentions it. The review was PROSPERO‐registered as a meta‐analysis but ended up as a narrative synthesis because of heterogeneity. Fair enough, that happens. But the abstract doesn't say so, and every effect estimate it reports therefore comes from a single study, not a pooled one. That matters, especially since Zhou et al.'s overlapping 14‐study meta‐analysis managed a pooled mortality estimate (HR 1.67, 95% CI 1.38–2.00) [2]. Was subgroup pooling by ejection‐fraction phenotype, say, or follow‐up length genuinely not feasible here, or just not attempted?

The headline numbers are the best‐case ones. The abstract leads with HR 3.28 for HFpEF mortality and OR 2.47 for 1‐month MACE, but doesn't mention that both come from small, single studies rated very‐low certainty; the MACE figure rests on just 96 patients, with a confidence interval (1.01–6.01) that barely clears the null. Meanwhile, the null results reported in the body of the paper 2‐month rehospitalization, AKI, eGFR, don't make it into the abstract at all. Boutron et al. gave this pattern a name in their landmark study of trial abstracts: spin [3].

The AUCs and the framing don't match. Reported AUCs of 0.56–0.62 are barely better than a coin flip, yet the Discussion still positions BCR as a promising low‐cost alternative to NT‐proBNP, troponin, and ST2. If BCR's clinical utility is going to be discussed, those AUC values deserve equal billing.

Some certainty ratings look inflated. Several Table 4 outcomes are graded moderate‐to‐high certainty despite resting on a single observational study which, under GRADE, starts at low certainty by default [4]. It would help to know explicitly which upgrading criteria were invoked (large effect? dose−response? confounding working against the association?).

Going forward, we'd like to see biomarker reviews report certainty‐weighted summaries directly in the structured abstract, and flag analytical‐plan deviations up front in line with PRISMA 2020 [5]. None of this undercuts the value of the review: BCR still looks like a promising, low‐cost marker of hemodynamic and renal stress. We just think its discriminative limits deserve the same prominence as its associations, since for most clinicians, the abstract is all they'll ever read.

We thank the authors and welcome continued dialogue.

## Author Contributions

The authors have read and approved the final version of this letter.

## Funding

The authors have nothing to report.

## Ethics Statement

The authors have nothing to report.

## Consent

The authors have nothing to report.

## Conflicts of Interest

The authors declare no conflicts of interest.

## Data Availability

Data sharing is not applicable to this article as no data sets were generated or analyzed during the current study.
